# Comprehensive phenomic and genomic studies of the species, *Pectobacterium cacticida* and proposal for reclassification as *Alcorniella cacticida* comb. nov

**DOI:** 10.3389/fpls.2024.1323790

**Published:** 2024-01-25

**Authors:** Joanna Jonca, Minna Pirhonen, Michal Mateusz Waleron, Jan Gawor, Agnieszka Mrozik, Magdalena Smoktunowicz, Krzysztof Waleron, Malgorzata Waleron

**Affiliations:** ^1^ Laboratory of Plant Protection and Biotechnology, Intercollegiate Faculty of Biotechnology, University of Gdansk and Medical University of Gdansk, Gdansk, Poland; ^2^ Department of Agricultural Sciences, University of Helsinki, Helsinki, Finland; ^3^ DNA Sequencing & Synthesis Facility, Institute of Biochemistry & Biophysics, Polish Academy of Sciences, Warsaw, Poland; ^4^ Institute of Biology, Biotechnology and Environmental Protection, Faculty of Natural Sciences, University of Silesia, Katowice, Poland; ^5^ Department of Pharmaceutical Microbiology, Medical University of Gdansk, Gdansk, Poland

**Keywords:** *Pectobacterium*, genomic studies, phylogenomics, PCWDEs, plant pathogen, phenotypic studies, FAME

## Abstract

**Introduction:**

*Pectobacterium cacticida* was identified as the causative agent of soft rot disease in cacti. Due to a high potential of spread in the face of global warming, the species poses a significant threat to horticultural and crop industry. The aim of this study was to revise the genomic, physiology and virulence characteristics of *P. cacticida* and update its phylogenetic position within the *Pectobacterium* genus.

**Methods:**

Whole genome sequences of five *P. cacticida* strains were obtained and subjected to comprehensive genomic and phylogenomic data analyses. We assessed the presence of virulence determinants and genes associated with host and environmental adaptation. Lipidomic analysis, as well as biochemical and phenotypic assays were performed to correlate genomic findings.

**Results:**

Phylogenomic analysis revealed that *P. cacticida* forms a distinct lineage within the *Pectobacterium* genus. Genomic evaluation uncovered 516 unique proteins, most of which were involved in cellular metabolism. They included genes of carbohydrate metabolism and transport and ABC transporters. The main differing characteristics from other *Pectobacterium* species were the lack of a myo-inositol degradation pathway and the presence of the malonate decarboxylase gene. All tested strains were pathogenic towards *Opuntia* spp., chicory, Chinese cabbage, and potato, but exhibited only mild pathogenicity towards carrot.

**Discussion:**

This study sheds light into the genomic characteristics of *P. cacticida* and highlights the pathogenic potential of the species. Unique genes found in *P. cacticida* genomes possibly enhance the species’ survival and virulence. Based on phylogenomic analyses, we propose the reclassification of *P. cacticida* to a new genus, *Alcorniella* comb. nov.

## 1 Introduction

The species *Erwinia cacticida* was founded in 1991 (Alcorn et al., 1991), and reclassified as *Pectobacterium cacticida* in 1998 (Hauben et al., 1998). They were identified to cause soft rot disease on saguaro, organ pipe, senita cacti, and Opuntia (Alcorn et al., 1991). The Gram-negative bacteria similar in characteristics to the soft-rot bacteria were isolated from saguaro cacti in the USA in 1961, and *Opuntia* spp. in Mexico in 1982 (Alcorn et al., 1991). In 1991, Alcorn described 88 bacterial cultures. The isolates were collected from the USA, Mexico, and Australia between 1958 and 1989 (Alcorn et al., 1991). The established species, *Erwinia cacticida* was described as consisting of Gram-negative, facultatively anaerobic rods with peritrichous flagella. They are catalase-positive and oxidase-negative, may produce capsules and are pectolytic. In addition, they produce acid from a number of carbohydrates (Alcorn et al., 1991).

*Pectobacteriaceae* have a high potential for spread in the environment and adaptation to different hosts (Perombelon and Kelman, 1980). To date, *P. cacticida* has not been reported in other countries than the USA, Mexico and Australia. However, its occurrence is possibly much broader as *Opuntia ficus-indica* (prickly pear), a commercially important crop which *P. cacticida* readily infects, is widely distributed around the world and common in Mediterranean, sub-tropical and tropical climates (Belviranlı et al., 2019). Moreover, *P. cacticida* was also isolated from sunflower (Valenzuela-Soto et al., 2015), which suggests that its host range may be broader than anticipated. As the global transmission of that species could cause substantial damage to the horticultural and crop industry, it is imperative to revise its characteristics and reassess its virulence potential. This phenomenon was already observed for other species of *Pectobacteriaceae* (Smoktunowicz et al., 2022).

Over the years, *P. cacticida* has been given relatively little attention. Only one genome of the type strain ATCC 49481 has been sequenced to date (Xie et al., 2023). The classification, physiology and virulence characteristics of these bacteria have not been revised and updated for over thirty years, since the original work of Alcorn et al. (1991). Recent analyses of the whole genome sequence of *P. cacticida* type strain ATCC 49481 revealed that *P. cacticida* represents the deepest basal branch of the genus *Pectobacterium*, and it has been suggested that the genus may require reclassification (Xie et al., 2023).

In this study, we present for the first time the whole genome sequences of five *P. cacticida* strains. The genomes were analyzed for the presence of virulence determinants and unique proteins that might shed light on the virulence potential of the species and their host adaptation capabilities. We correlated those findings with biochemical assays and pathogenicity tests to conffirm the activity of discovered genes. Based on the phenotypic, chemotaxonomic, as well as genomic and phylogenomic data, we suggest the reclassification of *Pectobacterium cacticida* into a new genus, *Alcorniella* comb. nov. within the *Pectobacteriaceae* family to accommodate strains currently classified as *Pectobacterium cacticida*.

## 2 Materials and methods

### 2.1 Bacterial strains

The *Alcorniella* comb. nov. strains used in this study are listed in **Table 1**, and the reference strains are presented in **Table S1**. The strains of various *Pectobacterium* species, *Samsonia erythrinae* CFBP5236 and *Chromobacterium violaceum* CV026 are a part of the *Pectobacterium* culture collection at the Intercollegiate Faculty of Biotechnology, Gdansk, Poland. All strains were stored in 40% glycerol at - 80°C and maintained on the Crystal Violet Pectate (CVP) medium (Perombelon and Burnett, 1991) at room temperature.

**Table 1:** *Alcorniella cacticida* strains used in this study.

IFB Collection Strain No.	Other Collections Designations	Strain Number^a^	Isolation Source	Region	Year of Isolation	RFLP group^b^
		66-93	*Carnegiea gigantea*	Arizona, USA	1967	
**IFB5353**	ICPB EC290	66-50	*Carnegiea gigantea*	Arizona, USA	1966	30
IFB5354		66-19-1	*Opuntia phaeacantha* var. *major*	Arizona, USA	1966	31
IFB5355	NCPPB 672	623-2	*Carnegiea gigantea*	Arizona, USA	1980	31
**IFB5356**	ICPB EC296; ICMP 7455	Texas 28	*Opuntia phaeacantha* var. *discata*	Texas, USA	1971	30
IFB5357		Texas 29	*Opuntia phaeacantha* var. *discata*	Texas, USA	1971	30
IFB5359		88-6	*Carnegiea gigantea*	Arizona, USA	1988	31
IFB5360		DU 89-7.3	*Opuntia stricta*	Australia	1989	31
**IFB5361**		DU 89-5.1	*Opuntia stricta*	Australia	1989	31
IFB5362	ICPB EC189	106	*Carnegiea gigantea*	Arizona, USA	1958	30
**IFB5363^T^**	CFBP3628^T^; ATCC 49485^T^; ICPB EC293	62-63	*Opuntia phaeacantha* var. *major*	Arizona, USA	1962	30
IFB5364	ATCC 49483	DU 89-8.1	*Opuntia stricta*	Australia	1989	30
**IFB5366**	CFBP 3222; ICMP 7452; ICMP 7452-81; NCPPB 3847	72-1	*Opuntia fulgida*	Arizona, USA	1958	29

a) (Alcorn et al., 1991).

b) (Waleron et al., 2002).

Strains selected for draft genome sequencing were marked with bold lettering.

### 2.2 Genetic characterisation of *Alcorniella cacticida*

The strains were grown overnight at 28°C on the Lysogeny Broth (LB) with 1.5% agar medium (Graso, Biotech, Poland). A single colony was picked up and grown overnight in 2 ml of LB medium at 28°C with shaking (120 rpm). Bacterial cells were harvested by centrifugation (5 min at 12 000 rpm). The DNA was extracted according to the cetyltrimethylammonium bromide (CTAB) protocol (www.jgi.doe.gov, accessed on 5 August 2023) and used as a template in PCR amplifications and DNA sequencing. The quantity and quality of the DNA were assessed by the spectrophotometric analysis with a microplate reader InfiniteM200Pro (Tecan, Männedorf, Switzerland) and the 1.5% agarose gel electrophoresis.

#### 2.2.1 DNA fingerprinting analyses

A total genomic DNA fingerprinting was performed by the repetitive element PCR fingerprinting (rep-PCR) using enterobacterial repetitive intergenic consensus (ERIC) primers (Versalovic, 1994). Analysis was performed with BioNumerics V6.6 (http://www.applied-maths.com). The UPGMA cladogram was created with a band base Jaccard coefficient.

#### 2.2.2 Genome sequencing, assembly and annotation

The genomes of four *A. cacticida* strains, IFB5353, IFB5361, IFB5163^T^, IFB5366 (**Table 1**) and *S. erythrinae* CFBP5236^T^ were sequenced using the Illumina MiSeq technology. Illumina compatible paired-end sequencing library was constructed using the NEB Ultra II FS Preparation Kit (New England Biolabs, Beverly, USA) according to the manufacturer’s instructions. The library was sequenced using an Illumina MiSeq platform (Illumina, San Diego, CA, USA) with 2 x 300 paired-end reads using v3 600-cycle sequencing kit. Sequence quality metrics were assessed using FASTQC (http://www.bioinformatics.babraham.ac.uk/projects/fastqc/). Quality and adapter trimming of the raw reads was performed using Trimmomatic v0.32 (http://www.usadellab.org/cms/?page=trimmomatic, accessed on 5 August 2023), kmer length and distribution were analysed using KmerGenie v1.6949 (http://kmergenie.bx.psu.edu/, accessed on 5 August 2023). *De novo* assembling was performed using SPAdes v3.10.1 (http://cab.spbu.ru/software/spades/, accessed on 5 August 2023), Velvet v1.2.10 (https://www.mybiosoftware.com/velvet-1-1-07-sequence-assembler-short-reads.html, accessed on 5 August 2023) and Ray v2.3.1 (http://denovoassembler.sourceforge.net/, accessed on 5 August 2023). The contigs were integrated using CISA v1.3 (http://sb.nhri.org.tw/CISA/en/CISA, accessed on 5 August 2023) software and scaffolded with SSPACE v3.0 (https://github.com/nsoranzo/sspace_basic, accessed on 5 August 2023). The final assembly was evaluated using Quast v4.5 (http://quast.sourceforge.net/quast, accessed on 5 August 2023) software. The strains IFB5356 and CFBP3628^T^ were additionally sequenced using the Oxford Nanopore platform. Prior to long-read library preparation, genomic DNA was sheared into ~30kb fragments using 26G needle, followed by size selection using a Short Read Eliminator kit (Circulomics, Baltimore, MD, USA). 5 µg of recovered DNA was taken for 1D library construction using SQK-LSK109 kit and 0,8 µg of the final library was loaded into R9.4.1 flow cell and sequenced on GridION sequencer (Oxford Nanopore Technologies, Oxford, UK). Raw nanopore data was basecalled using Guppy v5.0.7 in super accuracy mode (Oxford Nanopore Technologies, Oxford, UK). After quality filtering using NanoFilt (De Coster et al., 2018) and residual adapter removal using Porechop (https://github.com/rrwick/Porechop), the obtained dataset was quality checked using NanoPlot (De Coster et al., 2018). Long nanopore reads were then assembled in hybrid mode using Unicycler v.0.5.0 (Wick et al., 2017, https://github.com/rrwick/Unicycler, accessed on 5 August 2023)). The final assemblies were evaluated using Quast v4.5 (http://quast.sourceforge.net/quast, accessed on 5 August 2023) software. The remaining ambiguities in the genome assemblies were verified by the PCR amplification of DNA fragments, followed by Sanger sequencing with an ABI3730xl Genetic Analyzer (Life Technologies, Carlsbad, CA, USA) using BigDye Terminator Mix v. 3.1 chemistry (Life Technologies, Carlsbad, CA, USA). All possible sequence errors and miss-assemblies were manually corrected using Seqman software (DNAStar, Madison, WI, USA).

Annotation was performed by the NCBI PGAPpipeline (https://www.ncbi.nlm.nih.gov/genome/annotation_prok/, accessed on 5 August 2023) and RAST (Rapid Annotation using Subsystem Technology) (http://rast.nmpdr.org/, accessed on 5 August 2023). The genomic sequence was additionally annotated with BlastKOALA (KEGG Orthology And LinksAnnotation) (https://www.kegg.jp/blastkoala/, accessed on 5 August 2023).

#### 2.2.3 Overall genomic analyses, phylogenomics and comparative genomics

To evaluate the assignment of the studied strains to novel species, in silico DNA–DNA hybridization (*is*DDH) and the average nucleotide identity (ANI) were calculated between the genomes of *A. cacticida* and the genomes of the other type or reference strains of the members of *Pectobacterium* and *Samsonia* genera (**Table S2**). In silico DDH comparison was performed employing the Genome-to-Genome Distance Calculator (GGDC 2.0; http://ggdc.dsmz.de/distcalc2.php) (Auch et al., 2010), using the recommended BLAST+ alignment and formula 2 (identities/HSP length) (Goris et al., 2007). The calculation of the average nucleotide identity (ANI) was performed using the PyAni module (v0.2.10) (Pritchard et al., 2015) with MUMmer (v3.23) (Kurtz et al., 2004).

The phylogenomic analysis based on the core proteins sequence comparison was done using PhyloPhlAn computational pipeline (https://huttenhower.sph.harvard.edu/phylophlan, accessed on 4 August 2023). The core protein analysis was performed using the genomic sequences of other *Pectobacterium* members available on GenBank and the phylophlan library of 400 conservated proteins of Prokaryotes and Archea.

Comparative genomics was additionally performed with the use of Pathway Tools Software v27.0 (http://bioinformatics.ai.sri.com/ptools/, accessed on 14 August 2023) and a Bacterial Pangenome Analysis (BPGA) pipeline (https://iicb.res.in/bpga/, accessed on 13 September 2023). The obtained results were subjected to the BLAST analysis against the UniProt protein database (https://www.uniprot.org/, accessed on 13 September 2023). The visual comparison of genome homology was done with BRIG (BLAST Ring Image Generator) (http://sourceforge.net/projects/brig, accessed on 5 August 2023) with the default settings (Alikhan et al., 2011). *A. cacticida* CFBP3628^T^ was used as a reference for comparison with the novel genomes of *A. cacticida*. The genomes of *A. cacticida* CFCC10813 (CP109947) and *S. erythrinae* CFBP3652^T^ (SMBY00000000) have been included for genomic comparison.

The presence of ICE and IME elements in the *Alcorniella* comb. nov. genomes was investigated using ICEfinder: a web-based tool (https://bioinfo-mml.sjtu.edu.cn/ICEfinder/ICEfinder.html, accessed on 20 September 2023).

The biosynthetic gene clusters encoding secondary metabolites related to the synthesis of phytotoxins, antibiotics and antimicrobial compounds were predicted using antiSMASH 4.0 (Blin et al., 2023) (https://antismash.secondarymetabolites.org/#!/about, accessed on 20 September 2023).

### 2.3 Chemotaxonomic characteristics – fatty acid composition

Fatty acid methyl ester analysis (FAME) was performed according to the procedure described by (Sasser, 1990). Briefly, the whole-cell derived fatty acids were isolated directly from bacteria growing on Muller-Hinton II (MHII) plates (Graso, Biotech, Poland) for 4 days at 28°C. Strains used in the analysis were *A. cacticida* IFB5360, *A. cacticida* IFB5359, *A. cacticida* IFB5362, *A. cacticida* IFB5364, *A. cacticida* IFB5356, and *A. cacticida* IFB5353 as well as reference strains of *Pectobacterium* genera: *P. polonicum* DPMP315^T^, *P. punjabense* CFBP8604^T^, *P. wasabiae* CFBP3304^T^, *P. atrosepticum* CFBP1526^T^, *P. peruviense* IFB5232^T^, *P. zantedeschiae* 9M^T^, *P. betavasculorum* CFBP2122^T^, *P. carotovorum* CFBP2046^T^, *P. odoriferum* CFBP1878^T^, and *P. aroidearum* CFBP8168^T^. After saponification, methylation, extraction and washing according to the procedure of Sasser (Sasser, 1990), the obtained Fatty Acid Methyl Esters (FAME) were analyzed using a gas chromatography-FID detector (7820A GC System, Agilent Technologies Inc., Santa Clara, CA, USA) and an Ultra 2-HP capillary column (cross-linked 5% phenyl methyl silicone, 25 m, 0.22 mm id and 0.33 m film thickness). A standard No. 1200-A solution (MIDI Inc., Newark, NJ, USA) was used to calibrate the Gas Chromatography (GC). Qualitative and quantitative analyses of the fatty acids were performed using the TSBA library (ver. 6.2B) and Sherlock Microbial Identification System software (MIDI Inc., Newark, NJ, USA). All analyses were made simultaneously for the tested *A. cacticida* and reference strains.

### 2.4 Phenotypic characterisation

#### 2.4.1 Ability to use different organic carbon sources - BIOLOG assay

Biochemical tests were performed with the BIOLOG GENIII plates (Biolog Inc., Hayward, CA, USA) according to the manufacturer’s instructions, using inoculation fluid A. Briefly, overnight cultures of bacteria on MHII plates were resuspended in 0.85% saline solution. 150 μl of bacterial suspension were added into each of the microplate wells. The change of color of the wells was evaluated with the naked eye. The following strains were used in the study: *A. cacticida* CFBP3628^T^, *A. cacticida* IFB5353, *A. cacticida* IFB5356, *A. cacticida* IFB5359, *A. cacticida* IFB5361, *A. cacticida* IFB5363, *A. cacticida* IFB5364, *A. cacticida* IFB5366, *P. actinidiae* IFB5641^T^, *P. aquaticum* CFBP8637^T^, *P. aroidearum* CFBP8168^T^, *P. atrosepticum* CFBP1526^T^, *P. betavasculorum* CFBP2122^T^, *P. brasiliense* CFBP6617^T^, *P. carotovorum* CFBP2046^T^, *P. jejuense* 13-115, *P. odoriferum* CFBP1878^T^, *P. parmentieri* CFBP8475^T^, *P. parvum* IFB5220, *P. peruviense* IFB5232^T^, *P. polaris* NIBIO1006^T^, *P. polonicum* DPMP315^T^, *P. punjabense* CFBP8604^T^, *P. quasiaquaticum* IFB5686, *P. versatile* IFB5636^T^, *P. wasabiae* CFBP 3304^T^, *P. zantedeschiae* 9M^T^, *S. erythrinae* CFBP3652^T^.

#### 2.4.2 Pathogenicity tests

Pathogenicity of the selected *Alcorniella* comb. nov. strains and *S. erythrinae* CFBP3652^T^ was assessed for *Opuntia* sp., *Zantedeschia rehmannii, Spathipyllum* sp., turmeric (*Curcuma longa*), ginger (*Zingiber officinale*), carrot (*Daucus carota*), chicory (*Cichorium* spp.), Chinese cabbage (*Brassica rapa* subsp. *pekinensis*) and potato (*Solanum tuberosum*). With some modifications, the tests were performed as described previously (Lee et al., 2013; Smoktunowicz et al., 2022). Briefly, plant leaves and tuber slices were surface sterilised by soaking in 5% (v/v) sodium hypochlorite (NaOCl), and then rinsed thrice with distilled water. The surface of the leaves was additionally disinfected with 70% ethanol. Bacterial cultures were grown on CVP medium for 48 h, then harvested and resuspended in phosphate-buffered saline (PBS) to an approximate cell density of 10^8^ CFU mL^-1^. A small incision was made on each vegetable leaf using a pipette tip and 25 μl of each bacterial suspension was inoculated into the damaged section. Pipette tips containing 50 μl of each bacterial suspension were driven into the flesh of the surface disinfected potato, ginger, turmeric and carrot slices. The experiments were repeated three times, each with three replicate samples. The vegetables were placed in sterilised plastic boxes with sufficient moisture and stored at 30°C for several days. Vegetables inoculated with PBS were used as a negative control.

#### 2.4.3 PCWDEs, N-acyl homoserine lactone and siderophore production

Pectine depolymerase and cellulase activities were assessed as described by (Andro et al., 1984). Briefly, for polygalacturonase/pectate lyase assay, isolates were incubated on a solid M63 medium (Miller, 1972) containing polygalacturonic acid. After 48 h incubation at 30°C, plates were stained by flooding with 10% (w/v) copper acetate, which forms a blue complex with the polymer, leaving clear haloes around colonies that produce pectolytic enzymes. Cellulase activity was assessed on a medium containing 0.1% (w/v) carboxymethylcellulose (CMC). After 48 h of incubation at 30°C, the plates were stained with an aqueous 0.1% (w/v) Congo red solution for 1 h at room temperature and washed with 1 M NaCl. Cellulase-producing colonies formed “halo” zones. Protease, lipase and oligo-1,6-glucosidase activities were assessed on skimmed milk, egg yolk agar and starch agar, respectively (Tindall et al., 2014). Siderophore production was assessed with chrome azurol S (CAS) agar plate assay (Himpsl and Mobley, 2019). The diameter of “halo” zones around bacterial colonies was measured in each case. Experiments were performed in two repetitions and the means were calculated for each strain. Gelatinase production was assayed with the standard gelatin stab method (Tindall et al., 2014). Liquefication of the medium was assessed after one week of incubation. Malonate utilization as a sole carbon source was evaluated using the malonate broth (Tindall et al., 2014). A shift in the pH indicator color from green to dark violet indicated malonate utilization.

The capability of the strains to produce N-acyl homoserine lactone (AHL) was assessed using *Chromobacterium violaceum* CV026 as a biosensor as described by (Ravn et al., 2001). Briefly, tested strains were streaked on the LA plates and then incubated overnight at 30°C. After that time, *C. violaceum* CV2026 was streaked on the plates in parallel. Plates were then once again incubated at 30°C overnight. After that time, the plates were evaluated for the purple violacein pigment produced by *C. violaceum* in the presence of AHLs.

#### 2.4.4 Growth assays

The growth of *A. cacticida* IFB5353 strain as well as *Pectobacterium* strains: *P. zantedeschiae* 9M^T^ and *P. aroidearum* CFBP8168^T^ in a microtiter plate was determined by absorbance (OD) measurement at 600 nm each hour for 72 hours of incubation at 28°C using a microplate reader InfiniteM200Pro (Tecan, Männedorf, Switzerland). Metabolic activity was assessed by the polypyridyl complex of Ru(II) method as described in (Jońca et al., 2021). The following media were used: M63 medium supplemented with 0.2% glycerol and 0.4% PGA, and M63 medium supplemented with 10% potato extract. Plant extract was prepared as described in (Jonca et al., 2021).

Additionally, the growth of *A. cacticida* strains at different temperatures in Tryptic Soy Broth (TSB) medium (Graso, Biotech, Poland) was assessed. Absorbance (OD) was measured at 600 nm after 24 hours of incubation using a microplate reader InfiniteM200Pro (Tecan, Männedorf, Switzerland).

### 2.5 Statistical analyses

All experiments were performed in triplicate unless stated otherwise. Errors were calculated as the standard deviation of the mean. The statistical significance of the pathogenicity tests and phenotypic assays was assessed using independent Student’s t-test and one-way Analysis of Variance (ANOVA). *Post-hoc* analysis was performed using Student’s t-test with a base mean as a reference. Statistical significance was assumed if p-value < 0.05. The statistical analysis was done using R software for Windows version 4.1.2 (R Foundation, Vienna, Austria) and the following packages: ComplexHeatmap (Gu et al., 2016), ggplot2 (Wickham, 2009), and ggpubr (Kassambara, 2023).

## 3 Results

### 3.1 Phylogenetic analysis

**Figure 1:**
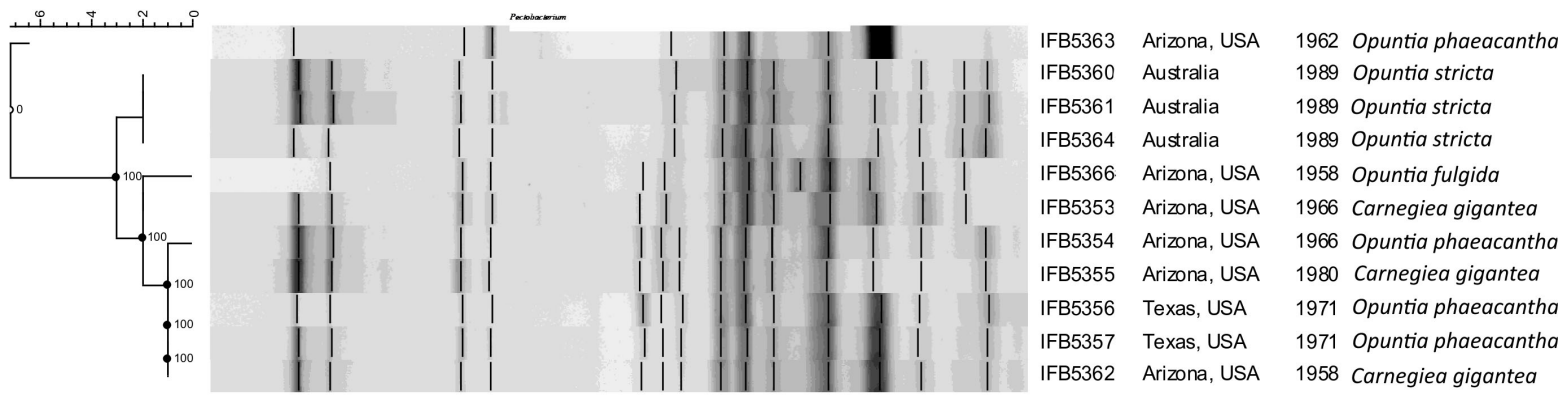
The rep-PCR fingerprint profiles obtained with ERIC primers. *A. cacticida* strains in the following order from top to bottom: 1 - 66-93-3; 2 - IFB5363; 3 - IFB5366; 4 - IFB5353; 5 - IFB5361; 6 - IFB5360; 7 - IFB5359; 8 - IFB5355; 9- IFB5364; 10 - IFB5354; 11 - IFB5362; 12 - IFB5357; 13 - IFB5356. The unweighted pair group method with arithmetic averages (UPGMA) cladogram was created with band base Jaccard coefficient, performed with using BioNumerics V6.6 (http://www.applied-maths.com).

The rep-PCR fingerprint profiles (**Figure 1**) obtained with the enterobacterial repetitive intergenic consensus (ERIC) primers revealed that the analyzed isolates are not clonal and belong to two distinct lineages. Strains of the first lineage (IFB5360, IFB5361, IFB5364) all originated from Australia and were isolated from Opuntia spp. The second lineage is more diverse and includes strains from Opuntia spp. and *Carnegiea gigantea*, all isolated in the USA.

**Figure 2:**
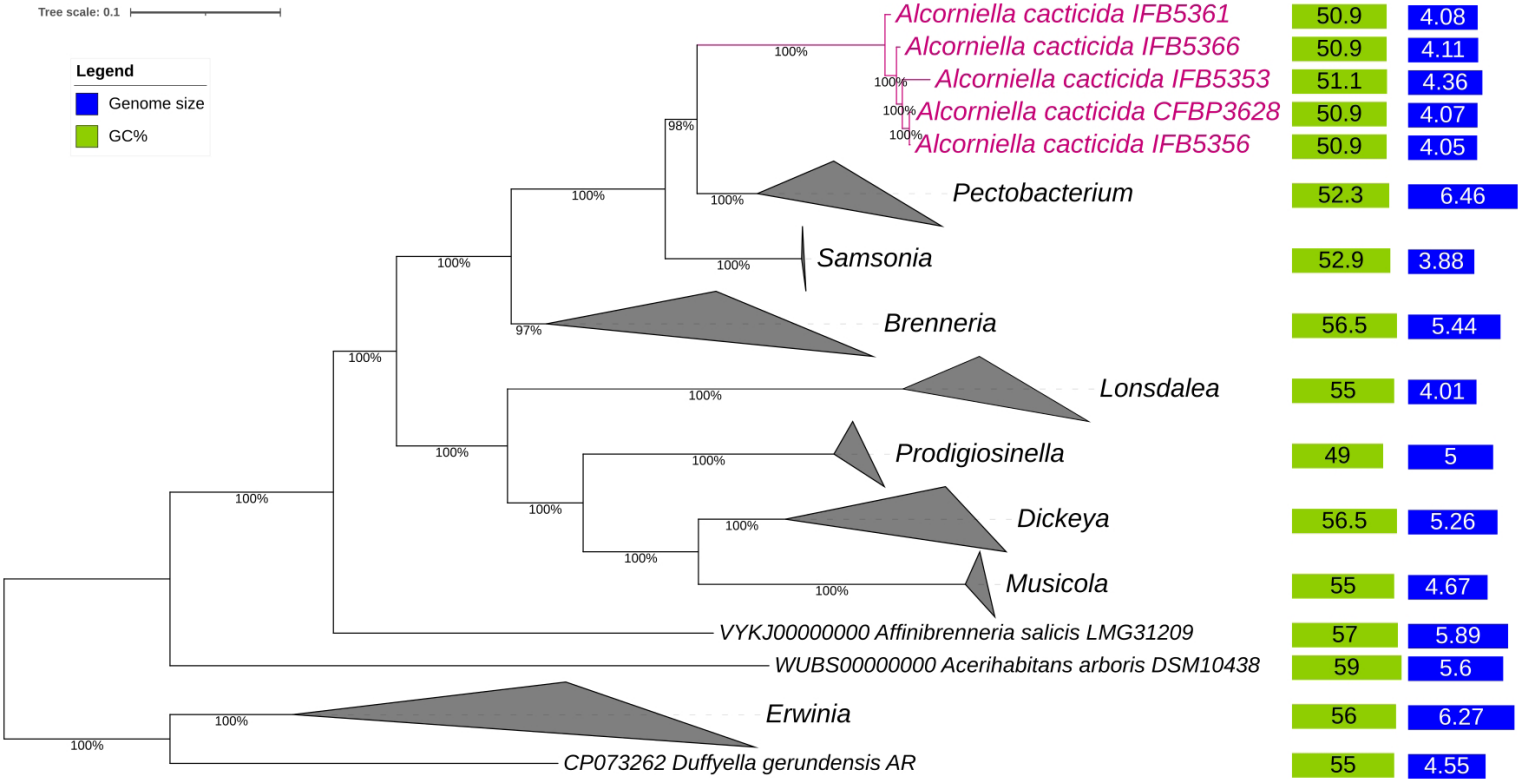
The phylogenomic analysis of *A. cacticida* strains based on the 400 most conserved universal proteins. The Maximum Likelihood tree was constructed using PhyloPhlAn computational pipeline (https://huttenhower.sph.harvard.edu/phylophlan, accessed on 7 August 2023).

The phylogenomic analysis of the whole genome sequences of *A. cacticida* strains based on the 400 most conservated protein sequences revealed that *A. cacticida* strains cluster together and form a separate phylogenetic lineage that is distinct from other species from *Pectobacterium* genus and known genera of *Pectobacteriaceae* family (**Figure 2**). *Alcorniella* comb. nov. branches at the root of the genus *Pectobacterium* and have a similar phylogenetic distance to those two families.

### 3.2 Genomic analyses

The 95-96% ANI threshold, corresponding to 70% DNA–DNA hybridization relatedness, has been recommended for species circumscriptions in prokaryotic taxonomy (Goris et al., 2007; Richter and Rosselló-Móra, 2009). The ANI values between the genomes of *A. cacticida* strains were above 97.58% threshold, confirming that they belong to the same species. Pairwise ANI values between the genomes of *A. cacticida* strains and the genomes of the representatives of *Pectobacterium* genus members were less than 84% (**Table 2** and **Supplementary Table 2**). It confirms that *A. cacticida* strains represent a novel genus.

**Table 2:** *is*DDH and ANI values between the proposed *Alcorniella* genus (in bold type) and related members of the *Pectobacteriaceae* family.

Genus	DDH	ANI
***Alcorniella cacticida***	**89.1-100**	**97.58-98.94**
*Pectobacterium*	25.7-27.7	82.10-83.54
*Samsonia*	23.8	80.37
*Brenneria*	21.1-21.6	75.96-77.01
*Dickeya*	19.7-20.8	73.57-74.54
*Musicola*	19.50-20.2	73.49-73.53
*Lonsdalea*	19.6-20.1	73.57-74.54
*Bistricola*	18.9	70.89

For isDDH calculation, A. cacticida type strain, CFBP3628^T^ was used as a reference genome.

*In silico* DNA–DNA hybridization (*is*DDH) is an in-silico method to replicate the wet-lab DDH method as closely as possible (Meier-Kolthoff et al., 2013). *is*DDH values were calculated between *Alcorniella* comb. nov. and related members of the *Pectobacteriaceae* family (**Table 2**). The species threshold was set to 70%. Pairwise calculated *is*DDH values between these genomes are consistent with ANI calculations. Pairwise *is*DDH values between the members of *Alcorniella* comb. nov. genomes were above 89.1%, well above the 70% species boundary. When pairwise calculations were performed between *Alcorniella* comb. nov. genomes and genomes of the *Pectobacterium* genus, the calculated pairwise *is*DDH values ranged from 25.7% to 27.7%, well below the 70% species boundary. Calculated pairwaise *is*DDH values between *Alcorniella* comb. nov. genomes and the genomes of other members of the *Pectobacteriaceae* family were below 25.7%. Those results suggest that *Alcorniella* comb. nov. should be considered as a genus separate from the *Pectobacterium* genus.

### 3.3 Whole genome sequencing

**Figure 3:**
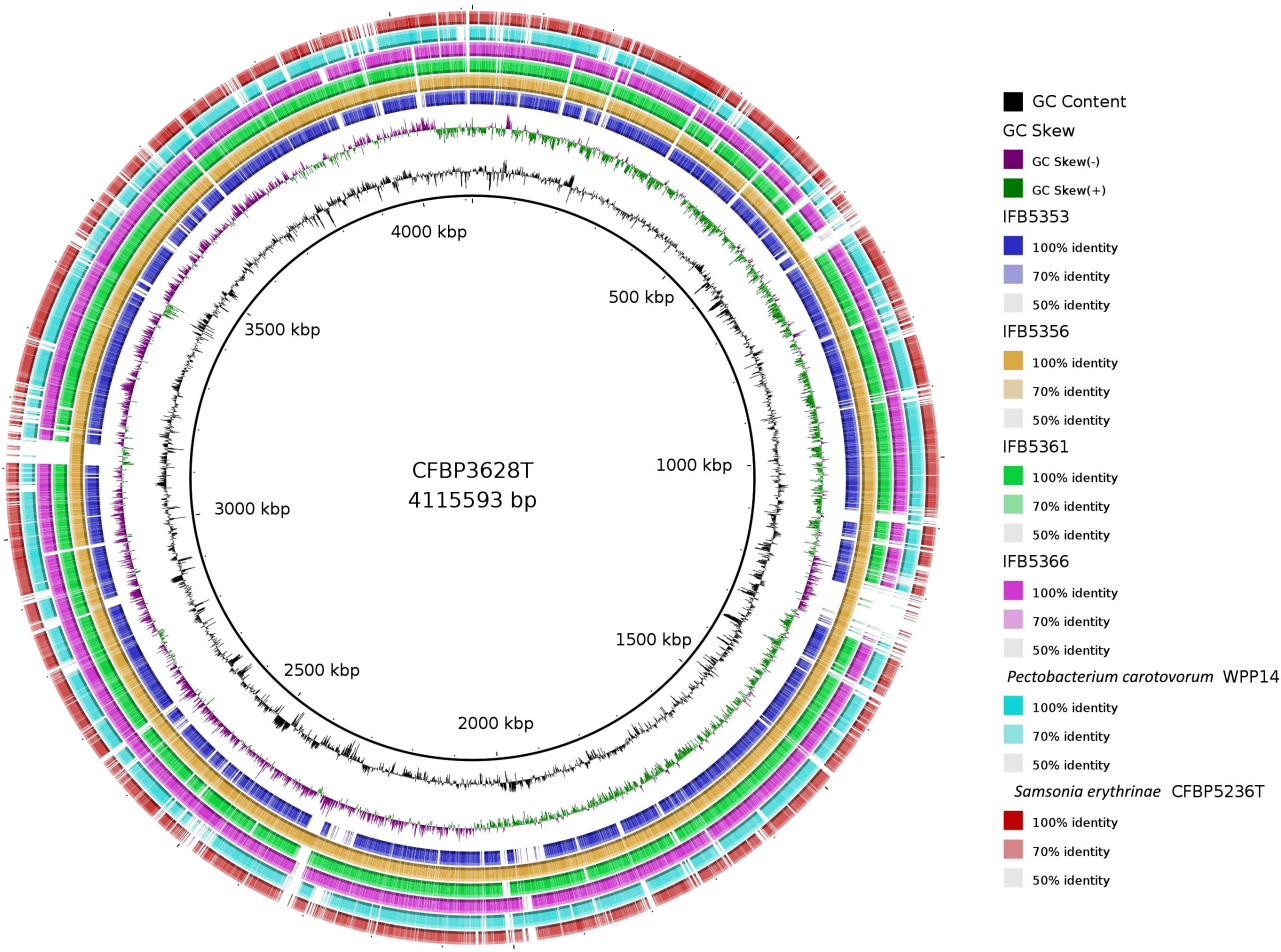
A graphical representation of the whole genome sequences of *A. cacticida* strains: CFBP3628^T^; IFB5356; IFB5356; IFB5366; IFB5361; IFB5353. The genomes of *Pectobacterium carotovorum* WPP14 and *S. erythrinae* CFBP5236^T^ were included for comparison. The map was created using BRIG platform application (Alikhan et al., 2011).

The genomes of five *A. cacticida strains* CFBP3628^T^, IFB5356, IFB5366, IFB5361 and IFB5353, and *Samsonia erythrinae* CFBP5236^T^ were sequenced, assembled, annotated, and deposited in GenBank under the accession numbers CP133656, CP125967, JASITJ000000000, JASITK000000000, LUCK00000000 and JAWIZJ000000000, respectively. The final genome sequences represent one chromosome each. The genome size was similar for the studied strains and was the smallest for *A. cacticida* IFB5356 (4.05 Mbp), and the largest for *A. caticida* IFBP5353 (4.37 Mbp). It did not substantially differ from the genome sizes of the members of *Pectobacterium* genus, which vary between 3.88 and 6.46 Mbp. The genome size of *Samsonia erythrinae* CFBP5236^T^ was 3.87 Mbp, and it was smaller than those for *Alcorniella* comb. nov. and *Pectobacterium.* The G + C content for sequences obtained from the draft genome was 51.1% for *A. caticida* IFB5353 strain and 50.9% for the other *A. caticida* strains. The results were in accordance with those for *Pectobacterium* genus (50.3-52.3%) and a bit lower than in the case of *Samsonia* (52.8%). The number of coding sequences (CDS) in the analyzed genomes was between 4030 and 4377. For *Pectobacterium* genomes, the number of CDS was between 3358-4210. There were between 67 and 74 RNA genes. No plasmids were found in the studied genomes. A graphical representation of the chromosome of *A. cacticida* CFBP3628 T and novel genomes of strains IFB5353, IFB5356, IFB5366, IFB5361 and *Samsonia erythrinae* CFBP5236^T^ was shown in **Figure 3**. Genome statistics were collected in **Supplementary Table 3**.

### 3.4 Comparative genomic analyses

**Figure 4:**
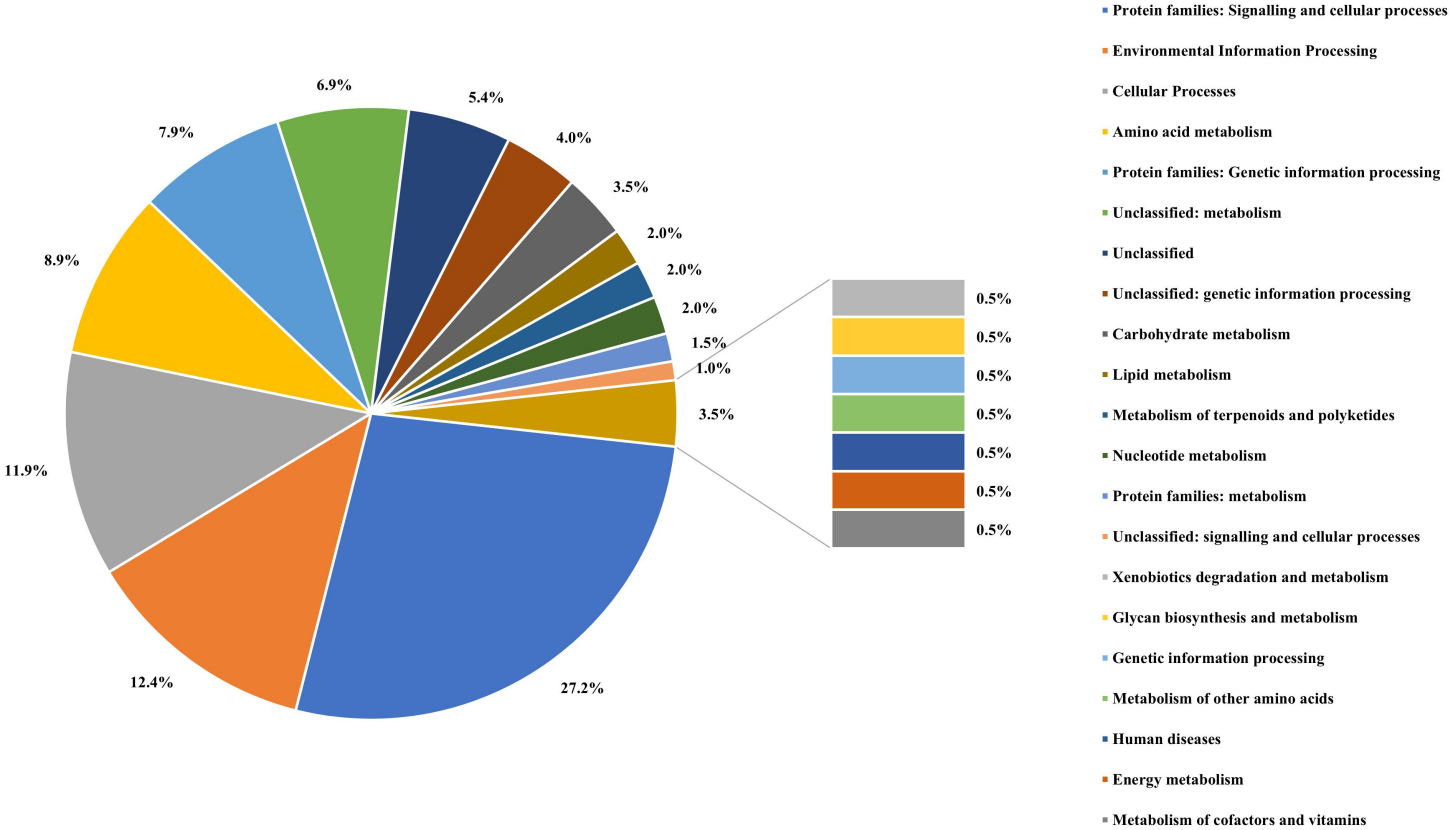
The percentage of the proteins assigned to each functional categories defined by the KEGG Orthology system for the result of BlastKOALA analysis performed on proteins unique for *A. cacticida* strains.

The assembled genome was annotated using the Rapid Annotation of the Subsystem Technology (RAST) server and COG (Clusters of Orthologous Groups). Genomes of *A. cacticida* strains were additionally annotated with the BlastKOALA. Comparative genomics analyses were also performed to further identify distinctive traits between *A. cacticida* strains and the members of *Pectobacterium* genus. The genome comparisons between the available genomes of *Pectobacterium* and novel genomes of *A. cacticida* strains yielded 516 unique proteins, out of which 38.8% were successfully annotated with BlastKOALA (**Figure 4**; **Supplementary Tables 4**, **5**). Most were assigned to the following functional categories: signaling and cellular processes (55), environmental information processing (25), cellular processes (24), amino acid metabolism (18), and genetic information processing (16). The majority of the coded proteins were involved in cellular metabolism, including carbohydrate metabolism, fatty acid biosynthesis, nucleotide and the amino acid metabolism of arginine, lysine and tyrosine. Multiple genes of ABC transporters were also present, as well as the genes of proteins involved in quorum sensing mechanism, urea and ferrum hydroxamate transport, and genes involved in the synthesis of rhamnolipids and siderophores such as enterochelin and pyochelin. Further analysis using the Pathway Tools software revealed the absence of 52 pathways in the genomes of *A. cacticida* strains in comparison to the *Pectobacterium* reference genomes (**Supplementary Table 6**). They involved a pathway for myo-inositol biosynthesis, 3-methylthiopropanoate biosynthesis, cis-vaccenate biosynthesis, erythritol biosynthesis, and glycine betaine degradation. The number of pathways in *A. cacticida* genomes that were absent in *Pectobacterium* was 36. The pathways involved xylan degradation, malonate utilization, quinate and shikimate degradation, and triclosan resistance.

**Figure 5:**
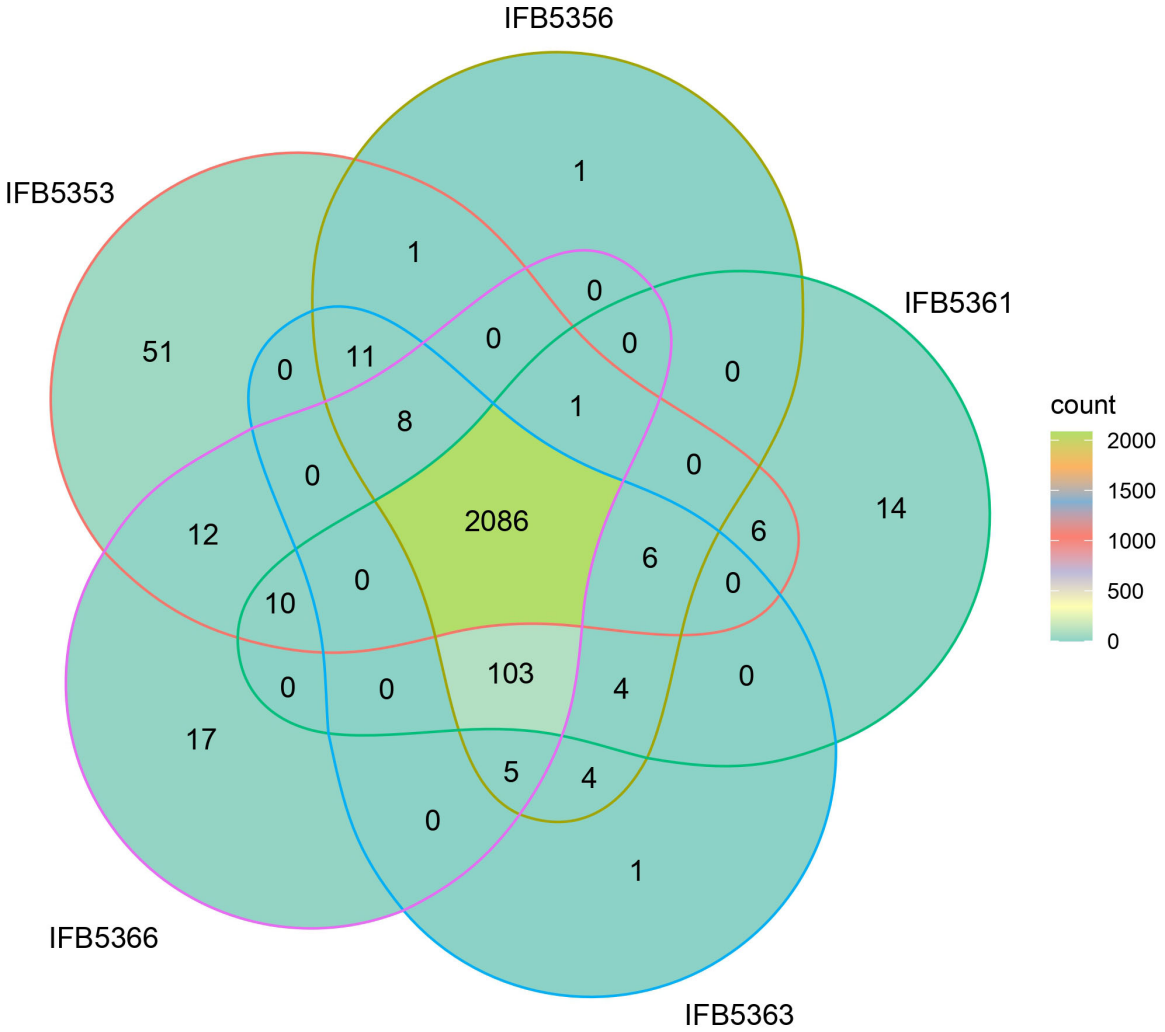
Venn diagram showing the result of BlastKOALA analysis performed on *A. cacticida* genomes.

There were 2066 core proteins present in *A. cacticida* genomes that were annotated with the Blast KOALA (**Figure 5** and **Supplementary Table 7**). Strain IFB5353, isolated from *Carnegiea gigantea*, had the most (51) additional genes. They involved genes of toxins and antitoxins such as *prlF-yhaV, hig*B-*hig*A *and fitA-fitB*, multidrug efflux systems and other transporters, and enzymes involved in fructose and mannose metabolism, amino sugar and nucleotide sugar metabolism and tyrosine metabolism. It also lacked the most genes (103) that were present in the other strains. They included genes of the type III secretion system, and genes involved in nitrogen, sulfur and butanoate metabolism. Several genes encoding ABC transporters were also not present, including genes of molybdate, taurine and ferric citrate transport systems.

Additional analysis using BPGA pipeline yielded similar results (**Supplementary Table 8**). The genome of IFB5353 strain differed the most from the genomes of the other strains in the number of additional genes present (480) as well as the number of genes absent (270) in its genome. The obtained gene sequences of genes uniquely absent in IFB5353 genome were further subjected to the BLAST analysis against the UniProt database (**Supplementary Table 9**). The genome lacked several genes involved in nitrogen metabolism, such as the gene of the molybdopterin-guanine dinucleotide biosynthesis protein MobC, and 5-methylthioadenosine deaminase.

Furthermore, the presence of genes encoding major virulence factors like Plant Cell Wall Degrading Enzymes (PCWDEs) and secretion systems was verified (**Supplementary Table 10**). Among PCWDEs in the *Alcorniella* comb. nov. genomes, there were seven genes encoding proteases, twenty-four pectin-degrading enzymes, two cellulose-degrading enzymes and five hemicellulose-degrading enzymes. Strains IFB5353 and IFB5361 lack one gene of rhamnogalacturonan hydrolase, and the gene of oligogalacturonate lyase. Strain IFB5361 additionally does not possess one of the pectate lyase genes (PelB), while strain IFB5366 does not have rhamnogalacturonan lyase gene.

Genetic determinants of the first (T1SS), second (T2SS), fifth (T5SS) and sixth (T6SS) secretion systems were detected in all five *A. cacticida* genomes (**Supplementary Table 11**). The third secretion system (T3SS) was present in all *Alcorniella* comb. nov. genomes except for the strain IFB5353. Genes encoding components of the fourth secretion system were present in the genomes of three *A. cacticida* strains (IFB5353, IFB5356, IFB5363^T^), but were not detected in the genomes of *A. cacticida* IFB5366 and IFB5361.

The genomes of *A. cacticida* strains were additionally screened for the presence of self-transmissible mobile elements such as integrative conjugative elements (ICEs) and integrative mobile elements (IMEs) that are known to affect the pathogenicity and lifestyle of phytopathogens (Gonçalves et al., 2022). Five different ICE elements were found, harboring genetic determinants of T4SS, and five putative IME elements (**Supplementary Table 12**). Integrative conjugative elements were present in all genomes except IFB5366, which harbored only one IME (IMEAca4). Two ICE elements, ICEAca1 and ICEAca2 were present in the genomes of the type strain CFBP3628^T^ and IFB5356. Three ICE and three IME elements were detected in the genome of the strain IFB5353. The ICEAca1 element harbors genes of Vir like T4SS, while ICEAca2 region harbors genes encoding type IV secretion pilus.

Subsequently, the presence of genetic determinants responsible for the synthesis of coronatine, siderophores, bacteriocins, signal molecules and other secondary metabolites produced non-ribosomally was verified in the genomes of *Alcorniella* comb. nov. (**Supplementary Table 13**). In the genomes of all *Alcorniella* comb. nov. strains, clusters of genes for the biosynthesis of secondary metabolites, such as 1-carbapen-2-em-3-carboxylic acid, O-antigen, siderophore (aerobactin), beta-lactone containing protease inhibitor (glidobactin) and homoserine lactone, were detected. *Alcorniella* comb. nov. strains differed in the composition of the gene clusters predicted to encode non-ribosomal peptides. Glycosylated lanthipeptide and nonribosomally synthetised metallophore (cahuitamycin) were only present in three strains: CFBP3628T, IFB5356, and IFB5366. In contrast, genomes of two strains, IFB5353 and IFB5361, had a gene of yersiniabactin. Linear peptides containing azol(in)e (klebsazolicin) were unique for strain IFB5366, while rhizomide A, xenoamicin A and bovienimide A biosynthetic gene clusters were unique for strain IFB5361. Notably, the genetic determinants responsible for coronatine synthesis were not detected in the *Alcorniella* comb. nov. genomes.

### 3.5 Chemotaxonomic characterisation - fatty acids composition

**Figure 6:**
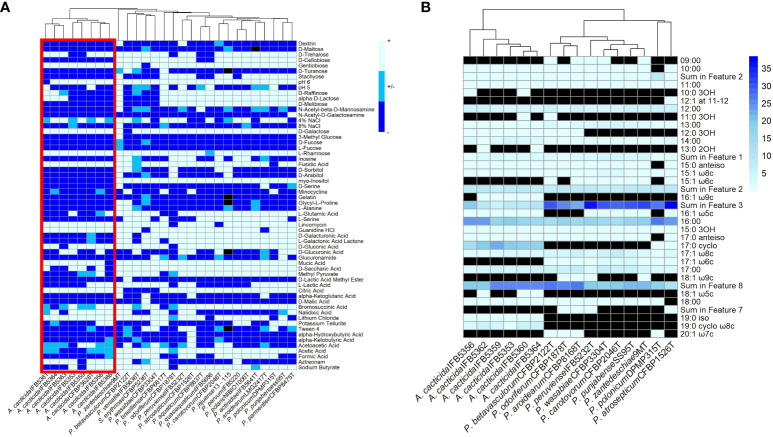
**(A)** Results of the BIOLOG assay with the strains panel of *A cacticida, Pectobacterium* genus reference strains and *Samsonia erythrinae* CFBP5236^T^. Only characteristics that differed between strains were shown. **(B)** The comparison of the total amount of fatty acids detected in six *A cacticida* strains (IFB5360, IFB5359, IFB5362, IFB5364, IFB5356, IFB5353) and ten *Pectobacterium* genus reference strains included for comparison. The values given are expressed as a percentage. Black color means absence of particular fatty acid.

The fatty acid analysis revealed the presence of 29 fatty acids (**Figure 6B**; **Supplementary Table 14**). The comparison of fatty acid content between representative *Pectobacterium* members showed that the profiles of the *A. cacticida* strains were mostly similar to the members of *Pectobacterium* genus. The predominant fatty acids that accounted for >5% of the total fatty acids in the studied *A. cacticida* strains were summed future 2, including 12:0 aldehyde (7-9%), summed future 3, including 16:1 ω7c and 16:1 ω6c (5-19%), 16:0 (15-23%), 17:0 cyclo (13-22%) and summed future 8, including 18:1 ω7c (19-25%). However, there was a difference in the content of 17:0 cyclo between *A. cacticida* strains (13-22%) and Pectobacteria (0-1%). *A. cacticida* strains also contained less summed future 3 fatty acids (including 16:1 ω7c and 16:1 ω6c) than Pectobacteria, 5-19% and 22-38%, respectively. The content of summed future 3 fatty acids varied between 5-10% for most of the *A. cacticida* strains, except for *A. cacticida* IFB5356, which contained significantly more (19%) of those fatty acids. There were also differences among the studied strains in the fatty acids, which accounted for less than 5% of the total fatty acids. Fatty acids 15:1 ω6c and 17:1 ω6c were absent in all of the studied *A. cacticida* strains, whereas they were detected in most of the tested Pectobacteria, 80% and 100%, respectively. In contrast, *A. cacticida* strains possessed 19:0 iso and 19:0 cyclo ω8c fatty acids, detected in none of the studied *Pectobacterium* strains.

### 3.6 Phenotypic characterisation

#### 3.6.1 Physiological characteristics

A total of 93 phenotypic characteristics were tested for eight *A. cacticida* strains and 20 reference strains, 19 reference strains belonging to the *Pectobacterium* genus and *S. erythrinae* type strain CFBP5236^T^. *A. cacticida* strains formed a distinct group from the other tested strains (**Figure 6** and **Supplementary Table 15**, **Supplementary Figure 2**). One significant difference was that none of the tested *A. cacticida* strains was positive for myo-inositol utilization, whereas all reference *Pectobacterium* strains could degrade that sugar. Another difference was the ability to grow at pH 6. All *Pectobacterium* reference strains were able to grow at pH 6, whereas *A. cacticida* strain IFBP5361 tested negative for growth at that pH. *A. cacticida* IFB5366 was negative for mucic acid utilisation, and four other *A. cacticida* strains were negative for D-saccharic acid utilisation while all tested *Pectobacterium* reference strains were able to utilise those compounds. *A. cacticida* strains were also negative or weakly positive for acetic acid utilisation, whereas all Pectobacterium reference strains were positive, except for one strain, P. odoriferum NCPPB3839T, which was weakly positive. In comparison, *S. erythrinae* CFBP5236^T^ differed from *Alcorniella* comb. nov. and *Pectobacterium* regarding the lack of gentobiose and guanidine utilisation, and the ability to utilise D-malic acid.

#### 3.6.2 Ability to macerate plant tissue

**Figure 7:**
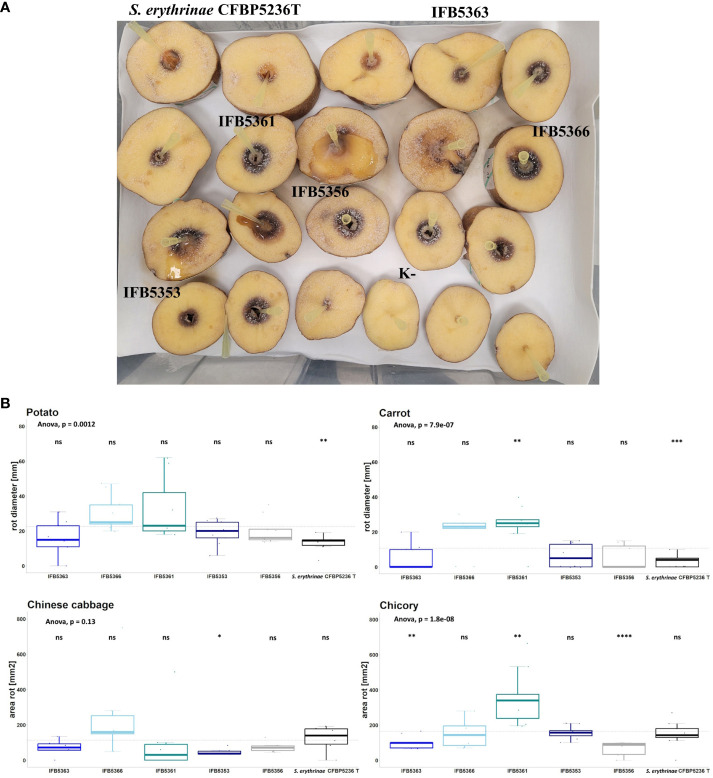
**(A)** The results of the virulence test on potato slices. The slices of potato tubers inoculated with *A cacticida* strains and *S. erythrinae* CFBP5236^T^ were incubated for 72 h at 30°C. After that time, the area of rotted tissue was measured. The order of strains from top left to bottom right: *S. erythrinae* CFBP5236 ^T^; *A cacticida* strains: IFB5363, IFB5361, IFB5366, IFB5356, IFB5353, K- (Negative control) **(B)** The results of pathogenicity tests. Values are means from three experiments with three replicate samples. The comparisons were made with a base mean as a reference, using Student’s t-test, ns – not significant, * p<0.5; ** p<0.1, ***p<0.01, **** p<0.001.

The virulence of strains selected for genome sequencing was tested against ornamental plants as well as crops. The results were presented in **Figure 7** and **Supplementary Table 16**. *A. cacticida* strains were pathogenic towards potato, Chinese cabbage, carrot, and chicory. All tested strains were also pathogenic towards Opuntia. Two strains, IFB5361 and IFB5366 exhibited the highest pathogenicity towards Opuntia; both have been isolated from this cactus. Only those strains were also pathogenic towards *Zantedeschia* sp. None of the tested strains caused disease symptoms on *Spathipyllum* sp., turmeric, and ginger root. One strain, IFB5361 was significantly more pathogenic towards carrot and chicory than the other strains.

#### 3.6.3 PCWDEs, siderophores and AHL production

The results of the enzymatic activity assays were listed in **Table 3**, **Supplementary Table 17**, and **Supplementary Figure 3**. All tested strains were able to degrade cellulose and pectates as well as produce siderophores and AHLs. One strain, *A. cacticida* IFB5356 was weakly positive for starch degradation. Two strains, IFB5353 and IFB5363^T^ were negative for casein degradation. All *A. cacticida* strains were able to utilize malonate as the only carbon source in contrary to *S. erythrinae*.

**Table 3:** The results of phenotypic tests.

Enzymatic activity assay	*A. cacticida* strains					S. *erythrinae* CFBP5236
	IFB5353	IFB5361	IFB5356	IFB5366	IFB5363	
Oligo-1,6-glucosidase	–	–	+/-	–	–	–
Beta-glucuronidase	+	+	+	+	+	+
Protease	–	+	+	+	–	+
Lipase	–	–	–	–	–	nd^a)^
Cellulase	+	+	+	+	+	–
Gelatinase	–	–	–	–	–	nd^a)^
AHLs production	+	+	+	+	+	+
Siderophore production	+	+	+	+	+	+
Malonate utilization	+	+	+	+	+	–

a) the test was not performed.

Selected strains of *A. cacticida* and *S. erythrinae* CFBP5236^T^ were assayed for the production of PCWDEs, AHLs and siderophores and utilization of malonate.

#### 3.6.4 Growth assays

The growth of the strain IFB5353, isolated from *C. gigantea*, was additionally assessed in M63 medium supplemented with 0.2% glycerol and 0.4% PGA, M63 medium supplemented with 10% potato extract, and compared with the growth of *Pectobacterium* reference strains in those media (**Supplementary Figure 4**). The metabolic activity of the strains was determined by the fluorescence measurements using polypyridyl complex of Ru(II) oxygen probe. All of the tested strains were able to grow in the M63 medium supplemented with 0.2% glycerol and 0.4% PGA and M63 medium supplemented with 10% potato extract. The IFB5353 strain had a similar OD after 72 hours of growth. However, the growth curve differed between those two media. In the M63 medium supplemented with 10% potato extract, the strain entered the logarithmic growth phase after 6 hours of incubation and reached the stationary phase after 13 hours of incubation. In comparison, in the M63 medium supplemented with 0.2% glycerol and 0.4% PGA, the strain entered the logarithmic phase of growth much later, after 26 hours of incubation and did not enter the stationary phase of growth during the measurement (72 hours). Overall, the IFB5353 strain exhibited similar growth characteristics to the *Pectobacterium* reference strains.

*A. cacticida* strains could grow over wide temperature ranges (**Supplementary Figure 5**), but temperature tolerance differed between strains. *A. cacticida* IFB5355, IFB5360, and IFB5363 exhibited only limited growth at 15°C. The optimal temperature of growth for all tested strains was between 27-37°C. All of the tested strains were able to grow at 47°C.

## 4 Discussion

The members of the genus *Pectobacterium* genus are highly heterogeneous (Adeolu et al., 2016). Formerly a part of *Erwinia*, the genus underwent extensive rearrangements over the years. Novel methods of genomic analyses based on whole genome sequencing became an essential tool in microbial studies. They improve the characterization of microorganisms and offer new insight into the mechanisms of bacterial pathogenicity. Moreover, they allow for better elucidation of complicated phylogenetic relationships between organisms than traditional approaches based on the phenotype characterization, 16S rRNA gene sequence or multi-locus sequence analysis (MLSA).

In this work, we characterized five new genomes of the strains previously identified as *Pectobacterium cacticida* species. Originally described by Alcorn et al. (1991), these bacteria differ in several characteristics from other members of *Pectobacterium* genus, such as the ability to grow at higher temperatures and improved resilience to drought. Earlier analyses based on the RFLP- PCR (Waleron et al., 2002) as well as 16S rRNA and MLSA analyses (Adeolu et al., 2016) placed *P. cacticida* at the deepest basal branch of the genus *Pectobacerium*. However, the lack of whole genome sequences of those bacteria made their proper classification difficult. Our phylogenomic analysis based on the 400 most conserved proteins as well as ANI and *is*DDH analyses revealed that the strains belonging to the species *A. cacticida* form a separate cluster, which branches at the root of *Samsonia* and *Pectobacterium*. Those results are in accordance with biochemical tests which confirm that they form a distinct group from the genus *Pectobacterium*. Therefore, we propose reclassifying the *P. cacticida* strains to the new genus, *Alcorniella* comb. nov.

The rep-PCR fingerprint analysis showed that *A. cacticida* strains form a diverse group and can be divided into two lineages depending on the origin of isolation. The results were consistent with the earlier RFLP – PCR results (Waleron et al., 2002). Genomic analysis revealed that *A. cacticida* possessed 516 genes annotated with KoalaBLAST that were absent in Pectobacteria. Overall, the additional genes were mainly involved in cellular metabolism and transport, and enhanced survival and pathogenicity of the bacteria. The additional genes included the *pep*E gene of peptidase E, which is a virulence factor enhancing colonization of the plant host (Lassy and Miller, 2000; Figueiredo et al., 2021). Creatinine deaminase gene *cod*A may enhance survival as creatinine is present in feaces of birds and animals and is a good nitrogen source (Bendt et al., 2004). Malonate decarboxylase decarboxylates the substrate to acetate and CO_2_ (Dimroth and Hilbi, 1997). It confers an additional advantage as malonate is present in plant tissues. It was shown that the ability to utilize malonate increases the virulence of *P. aeruginosa* (Elmassry et al., 2021). Phenotypic tests confirmed the activity of the gene in all tested *A. cacticida* strains. The result is consistent with the work of Alcorn (Alcorn et al., 1991), who indicated on the ability to utilize malonate as one of the key characteristics discerning *A. cacticida* from *Pectobacterium* genus. Other genes present encoded iron chelators. *A. cacticida* possesses a complete pathway for pyochelin synthesis. The ability to bind iron undoubtedly gives those bacteria a competitive advantage in desert environments deficient in this compound (Kaplan et al., 2021). What is more, they possessed a xylan degradation pathway. The ability to degrade xylan may play a major role in the virulence of the species, as the main polysaccharides in the callus of cacti are cellulose and xylan (Steelink et al., 1968). Quinate and shikimate are compounds present in decaying plant matter (Hawkins et al., 1985). The ability to degrade those compounds may be advantageous in the desert environment where energy sources are scarce. *A. cacticida* also possesses genes conferring triclosan resistance. Triclosan is a widely used biocide and is present in the environment (Wang and Liang, 2021), thus, the resistance genes may give those bacteria an advantage in highly polluted areas. The genomes were also analyzed for the absence of genes present in *Pectobacterium* genus that could potentially impact the virulence. *A. cacticida* could not utilize myo-inositol as the only carbon source as, contrary to Pectobacteria, the species lacks the genes for the degradation of this compound. It is consistent with the work of Alcorn et al. (1991). It was found that the ability to degrade myo-inositol enhanced the virulence of *Ralstonia solanacearum* and allowed colonization of plant roots (Hamilton et al., 2021). This ability is important for soilborne bacteria and probably would be of limited advantage in desert environments. They also lack the 3-methylthiopropanoate biosynthesis pathway. Its product, 3-methylthio-propionic acid is phytotoxic and affects plant leaves, causing wilting and chlorosis in plants (Lee et al., 2005). *A. cacticida* is also deficient in genes for *cis*-vaccenate biosynthesis. This compound is synthesized from palmitoleate at lower temperatures and plays an essential role in regulating membrane composition for adaptation to temperature changes (Dong and Cronan, 2021). It gives the bacteria the ability to grow over wide temperature ranges. The other pathways not found in *A. cacticida* genomes were erythritol biosynthesis and glycine betaine degradation, which play a role in osmoregulation (Wood, 2006; Janek et al., 2017). Growth assays confirmed that *Alcorniella* comb. nov. was able to grow at higher temperatures with the optimum between 27-37°C than *Pectobacterium* for which the optimal growth temperatures are between 26-34°C, depending on the species (Perombelon and Kelman, 1980). Fatty acid analysis revealed that *Alcorniella* comb. nov. strains did not differ significantly in fatty acid composition from *Pectobacterium* genus. However, *A. cacticida* possessed 17:0 cyclo and 19:0 cyclo ω8c fatty acids in contrast to Pectobacteria. This fatty acid group may serve a dual purpose in bacterial membranes (Poger and Mark, 2015). On the one hand, they stabilize membranes against unfavorable conditions through induction of a greater degree of order than their unsaturated precursors and restraint the rotation of the bonds surrounding the cyclopropane ring. Secondarily, they disrupt lipid packing, favor the occurrence of “gauche” defects in the fatty acid chains and increase the lateral lipid diffusion, consequently enhancing membrane fluidity. Here, cyclopropane fatty acids will likely increase the membrane order and sealing to prevent cations from entering the cell interior under changing temperature conditions (Nowak et al., 2021). Therefore, the presence of those fatty acids may allow a better survival in stressful environments, and they are an important factor in protecting the cells from chemicals and environmental factors such as temperature (Denich et al., 2003; Kaur et al., 2005). Undoubtedly, the presence of those fatty acids confers increased resilience of *Alcorniella* comb. nov. to harsh conditions of the desert.

We reevaluated *A. cacticida* strains phenotypic characteristics and virulence. Due to intense international trade in ware vegetables worldwide, the potential of spreading soft rot bacteria to new niches is high, especially in the era of global climate change. Moreover, *A. cacticida* can grow well over wide temperature ranges, which could further its spread. It is the more important as currently, there are no effective methods for controlling the soft rot disease (Mansfield et al., 2012). Identifying factors that improve the virulence and survival of *A. cacticida* is also of commercial importance as it is considered the best candidate for biofuel production from cacti (Blair et al., 2021). The results of virulence analysis confirm that *Alcorniella* comb. nov. has a broad host range, not limited to cacti. They are capable of infecting ornamental plants and important crops, such as potato and carrot, although their pathogenicity towards carrot is much weaker than towards potato, which is in accordance with the original work of Alcorn et al. (1991). Of the tested strains, CFBP5328^T^, isolated from Opuntia, and IFB5353, which was isolated from saguaro, had a markedly decreased virulence towards chicory in comparison to the other strains. Those strains were also unable to degrade casein. The growth of the *A. cacticida* type strain CFBP5328^T^ in various media as well as several *Pectobacterium* strains was characterized earlier (Jonca et al., 2021). The *A. cacticida* type strain differed from the tested *Pectobacterium* strains regarding its inability to degrade raffinose, which agrees with the results of the BIOLOG assay. Raffinose is not present in a significant amount in cacti (Stintzing and Carle, 2005); therefore, the ability to utilize that sugar would not provide an advantage to *A. cacticida*. In this work, we assessed the growth of IFB5353 strain in M63 medium supplemented with 0.4% PGA and 0.2% glycerol, a synthetic growth medium, and M63 media with addition of potato extract. The strain grew well in the M63 medium supplemented with 10% potato extract and in the synthetic M63 medium. Those results are in accordance with the pathogenicity tests, which showed that the strain could infect potato. The genome of IFB5353 strain differed the most from the other strains. Genome analysis revealed that its genome lacked 103 genes present in the other tested *A. cacticida* strains, including the absence of genes of type III transport system and several genes responsible for nitrogen metabolism. The strain had an additional 51 genes absent in the other tested *A. cacticida* strains. They included genes responsible for enhanced survival in the environment and the genes of tyramine oxidase (*tyn*A and *tyn*B), which are involved in the oxidative degradation of monoamines. Saguaro stems contain about 0.7% of alkaloids, including carnegine, gigantine and dopamine (Bruhn, 1971). The presence of these genes may play a role in providing an additional nitrogen source for the organism. Of the tested strains, the IFB5361 strain exhibited the highest virulence towards chicory and carrot. Comparison between its genome and the genome of the type strain IFB5363^T^ yielded 356 genes unique to *A. cacticida* IFB5361 of which 12% were annotated with BLASTKoala (**Table S18**). Amongst them were genes encoding quorum sensing proteins, virulence factors such as lysozyme, and genes conferring the ability to degrade lactose (β-galactosidase, *lac*Z) and terpenes (monoterpene ε-lactone hydrolase, *mlh*B). Degradation of terpenes plays an important role in the detoxification of plant secondary metabolites (Francoeur et al., 2020). Overall, the genes identified in *A. cacticida* IFB5361 strain may contribute to its increased virulence. Surprisingly, IFB5361 as well as IFB5366 lacked the type IV secretion system, but it did not negatively affect their virulence towards the tested plants. The type IV secretion system is involved in adhesion to the host and biofilm formation (Burdman et al., 2011). The strain genome possessed additional genes for the biosynthesis of secondary metabolites that were absent in the other *A. cacticida* genomes, all belonging to the non-ribosomal peptide synthetase type. Xenoamicin and rhizomide possess antimicrobial properties (Booysen et al., 2021; Tian et al., 2022), which undoubtedly aids in the competition with other microbes, whereas bovienimide A is interestingly believed to be involved in insect colonization (Li et al., 2021). It might suggest that some *A. cacticida* could be spread by insects, similar to *Pectobacterium* (Toth et al., 2021).

*A. cacticida* genomes were also analyzed for the presence of integrative conjugative elements (ICEs) and integrative mobile elements (IMEs). The sequence of *cfl* gene (KR018448.1) encoding coronafacic acid synthetase ligase from *P. cacticida* strain FHLGJ22 isolated from sunflower was not detected in any of the sequenced *Alcorniella* genomes. This observation contradicted to the results described by (Valenzuela-Soto et al., 2015). The protein sequence of Cfl was only 51% identical to the class I adenylate-forming enzyme family protein from *Brenneria tiliae* (WP_249244602.1) and in 44% similar to the coronafacate ligase in *P. atrosepticum* (ACX42385.1).

## 5 Conclusions

In this study, we presented novel whole genome sequences of five *A. cacticida* strains. We analyzed the genomes and found several virulence determinants and genes responsible for the adaptation to the host and environment. We correlated our findings with biochemical and pathogenicity studies and affirmed the virulence potential of the species. The genomic, phylogenomic, as well as phenotypic and chemotaxonomic analyses revealed that *A. cacticida* strains differed in several characteristics from *Pectobacterium* species. Therefore, we propose the reclassification of *Pectobacterium cacticida* based on a whole-genome-based phylogeny of five strains to a novel genus *Alcorniella* comb. nov.

## 6 Description of *Alcorniella* comb. nov.

*Alcorniella* comb. nov. (Al.corn.i.el’la. N.L. fem. *Alcorniella*, in reference to the former plant pathologist S. M. Alcorn, who described the species *Erwinia cacticida*).

Members of the genus are Gram-negative motile, non-sporulating rods with peritrichous flagella, catalase positive, oxidase negative, nitrate-reducing, facultatively anaerobic, and pectinolytic, producing pits on crystal violet pectate (CVP) medium. The genomic DNA G+C content is 50.9-51.1 mol%. The genus is a member of the family *Pectobacteriaceae* of the order *Enterobacterales* in the class Gammaproteobacteria. The five sequenced strains of the genus *Alcorniella* belong to the species *A. cacticida*.

All tested strains of the genus *Alcorniella* are able to utilize gentiobiose, sucrose, β-Methyl-D-glucoside, D-salicin, N-acetyl-D-glucosamine, α-D-dlucose, D-mannose, D-fructose, D-galactose, L-rhamnose, D-mannitol, glycerol, D-Glucose-6-PO_4_, D-Fructose-6-PO_4_, D-aspartic acid, L-aspartic acid, citric acid, and L-malic acid. All are resistant to troleandomycin, guanidine HCl, rifamycin, lincomycin, vancomycin, and grow in 1% sodium lactate, fusidic acid, tetrazolium salts, niaproof 4 and salinity up to 4% NaCl. All strains of the genus are unable to utilise dextrin, D-maltose, D-cellobiose, D-turanose, stachyose, D-mellibiose, N-acetyl-D-mannosamine, N-acetyl-D-galactosamine, N-acetyl-neuraminic acid, D-methyl-glucose, D-fucose, L-fucose, inosine, D-sorbitol, D-arabitol, myo-Inositol, D-galacturonic acid, L-galactonic acid lactone, glucuronamide, quinic acid, p-hydroxyphenylacetic acid, D-lactic acid methyl ester, α-ketoglutaric acid, D-malic acid, γ-aminobutyric acid, α-hydroxybutyric acid, β-hydroxy-D, L-butyric acid, α-ketobutyric acid, propionic acid, acetic acid, formic acid. They can also not use serine, L-alanine, L-arginine, glycyl-L-proline, L-histidine, L-pyroglutamic acid and degrade gelatin and Tween 40.

The species *A. cacticida* demonstrates the highest proportions of fatty acids 16:0 (15-23%), 17:0 cyclo (13-22%) and summed future 8, which included 18:1 ω7c (19-25%) fatty acids.

## Data availability statement

The original contributions presented in the study are included in the article/**Supplementary Files**. Further inquiries can be directed to the corresponding authors.

## Author contributions

JJ: Data curation, Formal analysis, Investigation, Visualization, Writing – original draft, Writing – review & editing. MP: Resources, Writing – review & editing. MMW: Data curation, Formal analysis, Validation, Writing – review & editing. JG: Formal analysis, Writing – review & editing. AM: Formal analysis, Writing – review & editing. MS: Formal analysis, Writing – review & editing. KW: Conceptualization, Formal analysis, Methodology, Project administration, Resources, Writing – review & editing. MW: Conceptualization, Data curation, Formal analysis, Funding acquisition, Investigation, Methodology, Project administration, Resources, Supervision, Visualization, Writing – review & editing.
