# Supplementary material for: Comprehensive phenomic and genomic studies of the species, Pectobacterium cacticida and proposal for reclassification as Alcorniella cacticida comb. nov
Source: Front Plant Sci. 2024 Jan 25;15:1323790. doi: 10.3389/fpls.2024.1323790 (PMC10850344; doi:10.3389/fpls.2024.1323790)
Supplement: Supplementary file 1 [file DataSheet_1.zip › Supplementary figures.docx]

Supplementary Material

# Supplementary Figures and Tables

## Supplementary Figures


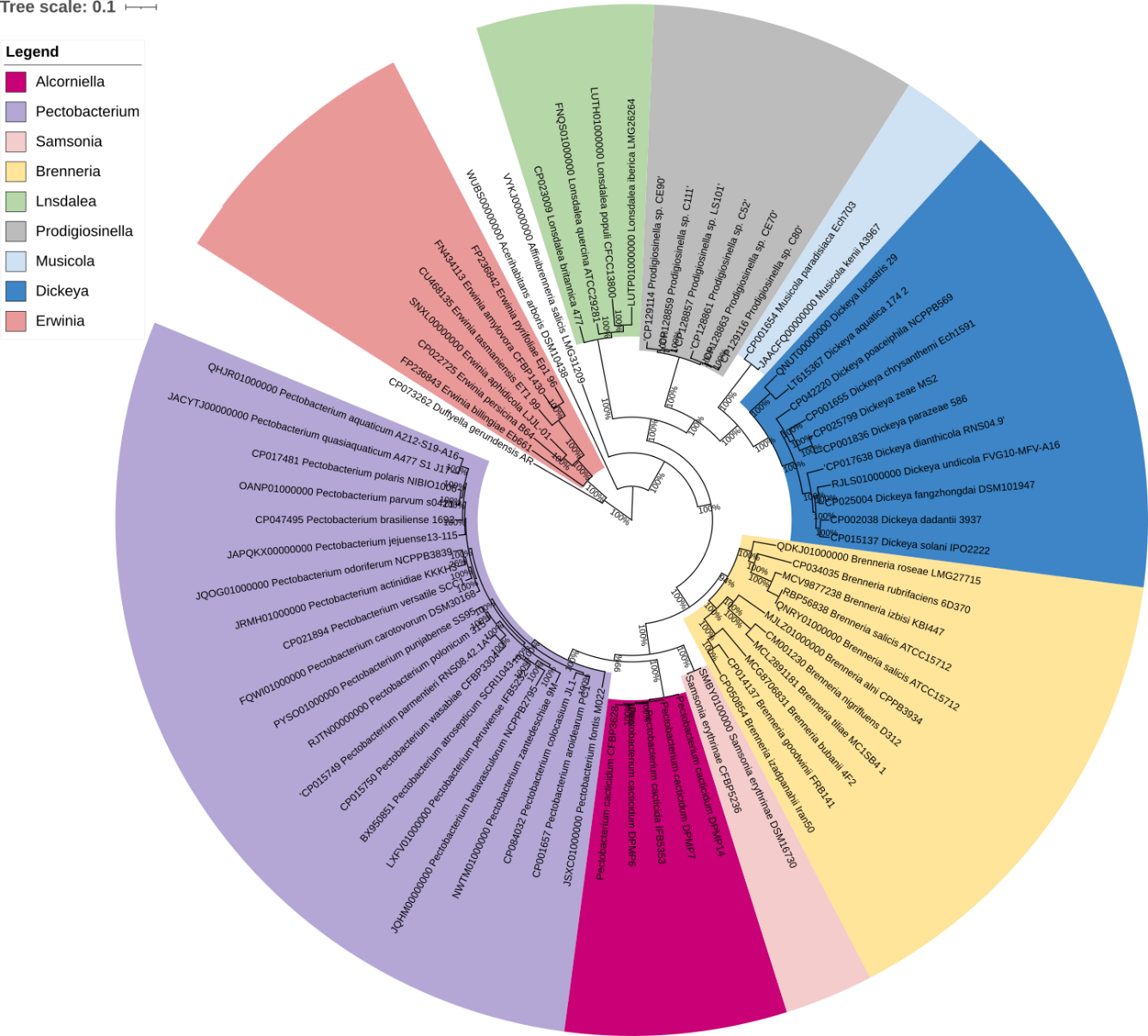


**Supplementary Figure 1.** The phylogenomic analysis of *A. cacticida* strains based on the 400 most conserved universal proteins. The Maximum Likelihood tree was constructed using PhyloPhlAn computational pipeline (<https://huttenhower.sph.harvard.edu/phylophlan>, accessed on 7 August 2023).


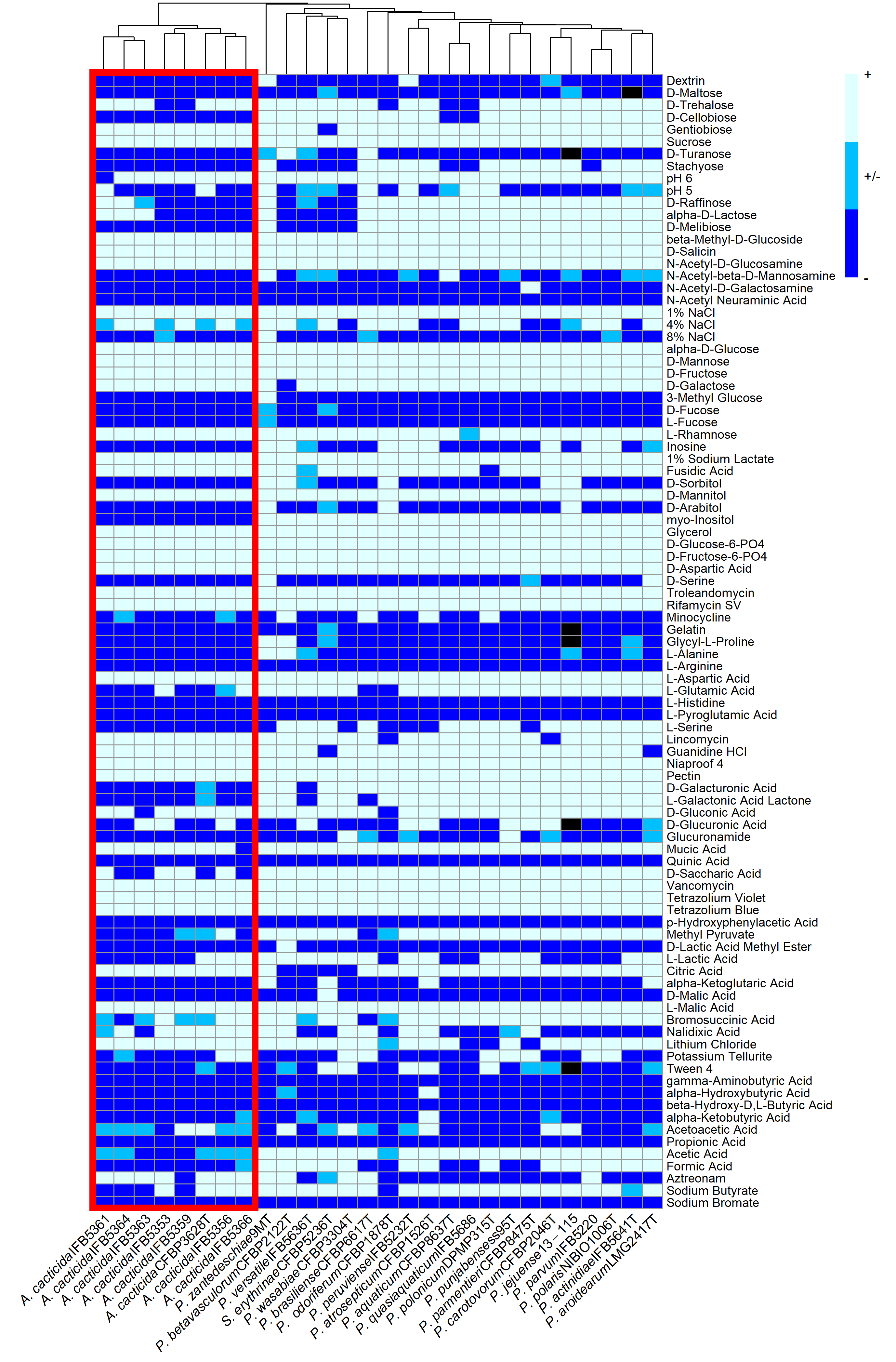


**Supplementary Figure 2.** Results of the BIOLOG assay with the strains panel of *A. cacticida*, *Pectobacterium* genus reference strains and *Samsonia erythrinae* CFBP5236^T^.


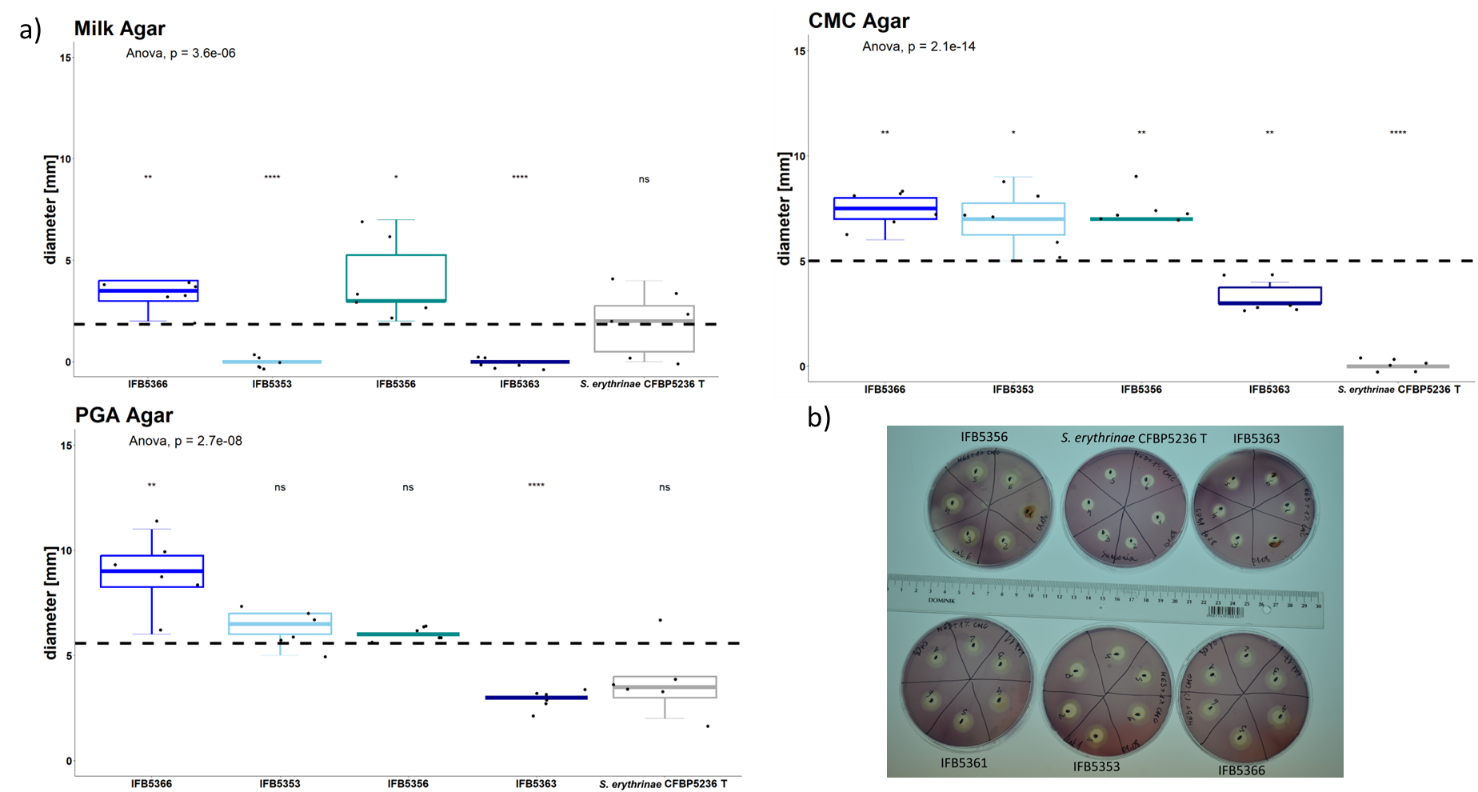


**Supplementary Figure 3.** The results of the phenotypic assays for *A. cacticida* strains and *S. erythrinae* CFBP5236^T^. **a)** Box plots depicting the results of the phenotypic tests. The comparisons were made with a base mean as a reference, using Student’s t-test, ns – not significant, * p<0.5; ** p<0.1, ***p<0.01, **** p<0.001. **b)** Cellulase assay results on CMC agar. The yellow “halo” zone around bacterial colonies indicates cellulose degradation.


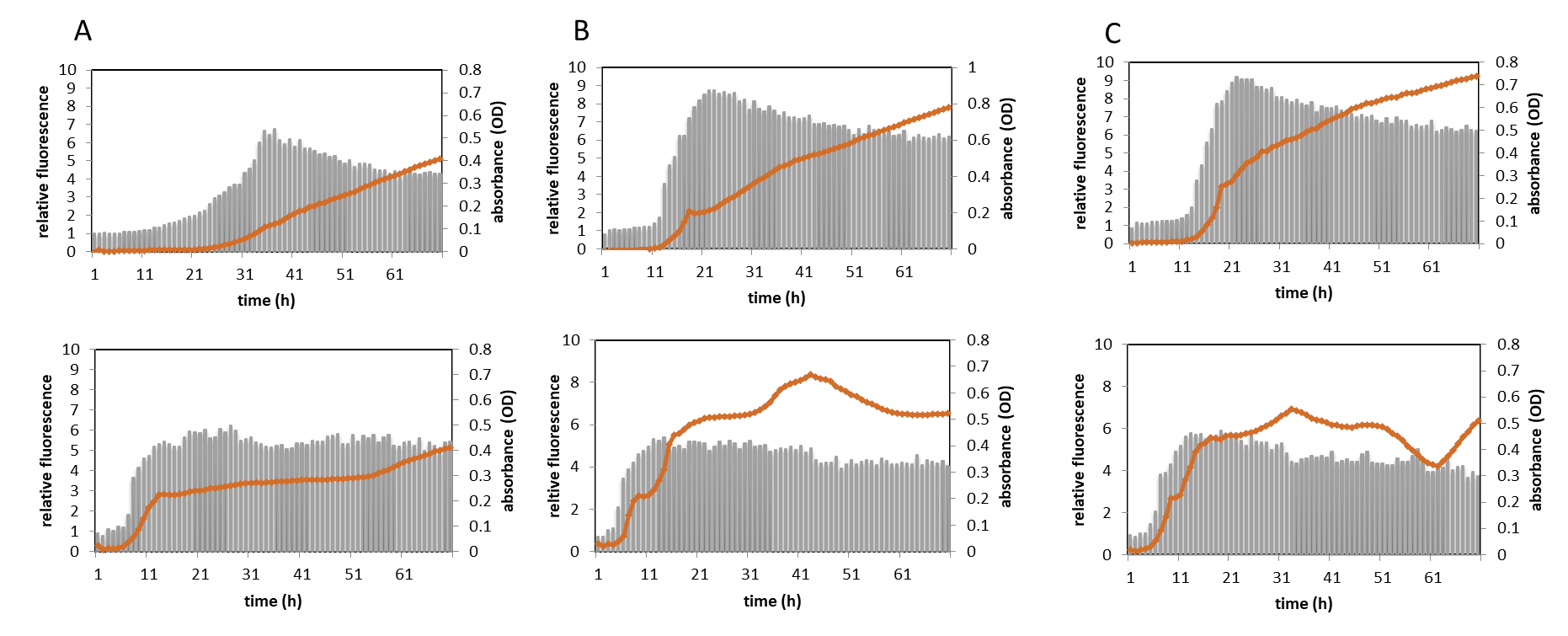


**Supplementary Figure 4.** Growth curves of a) *A. cacticida* IFB5353, b) *P. aroidearum* CFBP8168^T^, and c) *P. zantedeschiae* 9M^T^ in two different media (from top to bottom): M63 supplemented with 0.4% PGA and 0.2% glycerol, and M63 medium supplemented with 10% potato extract.


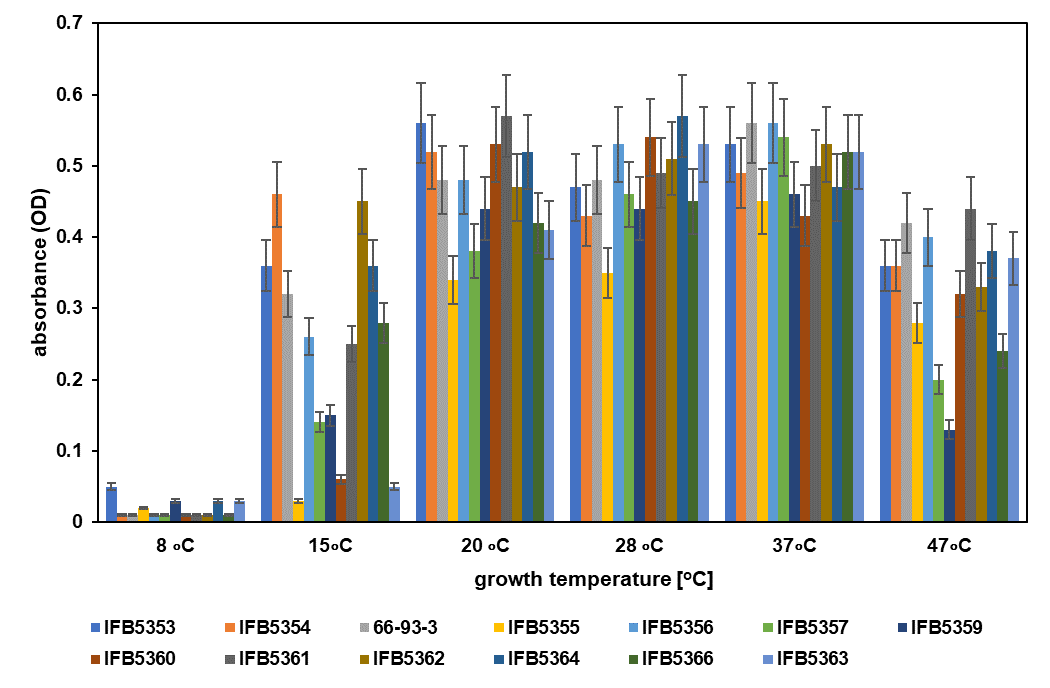


**Supplementary Figure 5.** Growth of *A. cacticida* strains in TSB medium at different temperatures after 24 hours of incubation.
